# Object of Eating

**Published:** 1884-11

**Authors:** 


### OBJECT OF EATING.

WE eat for warmth and strength ; hence almost all articles of food have both these elements ; have carbon to warm and nitrogen to strengthen, to give power to work. Butter, sugar and oils are almost all carbon. All breads and grains are mainly carbon. Meats, flesh of all kinds, abound in nitrogen. Food which has most nitrogen is most "nutritious."

Butter has eighty-three per cent. of carbon and no nitrogen ; an egg has no carbon and twenty per cent. of nitrogen. Milk contains two parts of warmth and one of strength. Bread con ains one part of nitrogen and eight of carbon. It is thus seen that in reference to eating, carbon—which is charcoal fuel and warmth, are one and the same thing; while nitrogen, which is, in effect, saltpetre—gives flesh or muscle, which are one and the same thing in substance, with strength. It is also seen that most articles of food have more carbon or warmth than nitrogen or strength, showing that it takes more to keep us warm than it does to keep us strong. A sedentary person requires, in round numbers, about one pound of food a day, while a hard-working man requires two pounds: this two pounds of food gives out power enough—as steam in an engine gives out power—to raise a man of average weight eleven miles high. But calling the two pounds 5000 grains, only 300 grains of it are nitrogen, and the remainder, carbon; that is, sixteen times more of warmth is required than of strength-producing food.

One practical result is, that as the world becomes more thickly populated, the necessity increases of economizing food ; of adapting it to the various needs of the system as modified by age, sex, occupation and season. Persons living in-doors, should not eat more than half as much as those who work hard. Less warming food should be eaten in hot weather than in cold. If we eat an excess of warming food in hot weather we have to work it out of the system at a great expenditure of strength ; and until it is worked off, we feel full and feverish, and oppressed; on the other hand, in winter we require an additional quantity of warming food, hence our instincts lead to eating heartily of pork, and buckwheat cakes, and butter, and molasses, which are almost purely carbon. In warm weather we need cooling food, and Providence sends us in profusion the fruits, and the berries, and the green things, which have no carbon at all; and while our appetite for them is ravenous, the very idea of fatty food is nauseating. Thus does the Beneficient One guide us, through our instincts, and provide wisely for the wants and needs of the system, according to the season, from infancy to age.

